# Plateau in Core Temperature during Shorter but Not Longer Work/Rest Cycles in Heat

**DOI:** 10.3390/ijerph21030371

**Published:** 2024-03-20

**Authors:** Joseph P. Bachraty, JianBo Qiao, Elizabeth S. Powers, Lesley W. Vandermark, J. Luke Pryor, Riana R. Pryor

**Affiliations:** Center for Research and Education in Special Environments, Department of Exercise and Nutrition Sciences, University at Buffalo, Buffalo, NY 14214, USAjianboqi@buffalo.edu (J.Q.); epowers2@buffalo.edu (E.S.P.);

**Keywords:** heat strain, work-to-rest ratio, intermittent work, hot environment, hyperthermia

## Abstract

This study compared physiological responses to two work/rest cycles of a 2:1 work-to-rest ratio in a hot environment. In a randomized crossover design, fourteen participants completed 120 min of walking and rest in the heat (36.3 ± 0.6 °C, 30.2 ± 4.0% relative humidity). Work/rest cycles were (1) 40 min work/20 min rest [40/20], or (2) 20 min work/10 min rest [20/10], both completing identical work. Core temperature (T_c_), skin temperature (T_sk_), heart rate (HR), nude body mass, and perception of work were collected. Comparisons were made between trials at equal durations of work using three-way mixed model ANOVA. T_c_ plateaued in [20/10] during the second hour of work (*p* = 0.93), while T_c_ increased in [40/20] (*p* < 0.01). There was no difference in maximum T_c_ ([40/20]: 38.08 ± 0.35 °C, [20/10]: 37.99 ± 0.27 °C, *p* = 0.22) or end-of-work T_sk_ ([40/20]: 36.1 ± 0.8 °C, [20/10]: 36.0 ± 0.7 °C, *p* = 0.45). End-of-work HR was greater in [40/20] (145 ± 25 b·min^−1^) compared to [20/10] (141 ± 27 b·min^−1^, *p* = 0.04). Shorter work/rest cycles caused a plateau in T_c_ while longer work/rest cycles resulted in a continued increase in T_c_ throughout the work, indicating that either work structure could be used during shorter work tasks, while work greater than 2 h in duration may benefit from shorter work/rest cycles to mitigate hyperthermia.

## 1. Introduction

Workers exposed to high ambient temperatures during prolonged heavy-intensity work are predisposed to increases in core temperature (T_c_) [1,2]. Excessive hyperthermia (T_c_ ≥ 38 °C) reduces worker productivity [3,4] while increasing the risk of negative health consequences such as heat illnesses [5,6], workplace injury [7,8], and cardiovascular events [9,10]. According to the Bureau of Labor Statistics, there were 43 work-related deaths in the United States due to environmental heat exposure in 2022, up from 36 deaths the previous year [11], and 4910 nonfatal occupational injuries and illnesses that resulted from exposure to heat from 2021 to 2022 [12]. Extreme heat events are projected to increase in frequency, intensity, and duration, placing workers worldwide at increased health risk for the foreseeable future [13]. To avoid these negative public health consequences, worker safety organizations provide recommendations for implementing work/rest cycles in hot environments based on the wet-bulb globe temperature, clothing worn, and estimated work intensity, with a goal of allowing for a plateau in T_c_ below 38.0 °C [14,15] to prevent excessive hyperthermia.

Heavy-intensity physical work in hot environments dramatically increases metabolic rate which leads to body heat production [1]. Despite the activation of heat loss mechanisms, an accumulation of body heat can occur during prolonged work, resulting in an increase in T_c_ [1]. Intermittent rest should be implemented during work to reduce heat production while encouraging heat dissipation via the continued evaporation of accumulated sweat on the skin surface. If mean arterial pressure is maintained, passive body cooling can be effective, resulting in a cooling rate of up to 0.07 °C·min^−1^ in ideal conditions [16,17]; however, some occupational settings do not allow for ideal body cooling during rest due to resting in warm to hot environments such as during agricultural work, roofing, or construction. Therefore, it is imperative that the structure of rest breaks encourages optimal heat dissipation, and that employers mandate or encourage rest breaks during physical work in the heat.

Few studies have directly explored work/rest cycle recommendations for workers during heat stress. Meade et al. [18] examined physiological responses to moderate intensity work in hot conditions, comparing 2 h of continuous work (41 °C, 19% relative humidity (RH)) and work/rest cycles of 15/5 min (43 °C, 17% RH), 15/15 min (46 °C, 13.5% RH), or 15/45 min (46.5 °C, 17.5% RH). T_c_ was greater at the end of continuous work and the 3:1 work/rest cycles, indicating that longer durations of rest compared to work durations mitigates increases in T_c_. However, each of these work structures took place in different environmental conditions as recommended by the American Conference of Governmental Industrial Hygienists (ACGIH) Threshold Limit Values (TLVs), allowing for practical determination of efficacy of the recommendations, but creating difficulty comparing the impact of work/rest cycles on physiological responses in the heat [19]. Additionally, the majority of participants were projected to have T_c_ ≥ 38 °C upon completion of 4 h of work when working continuously or in accordance with 3:1 or 1:1 work-to-rest ratios. These outcomes were also reflected in a comparable study of older workers [20]. Similarly, Mulholland et al. [21] determined that cardiovascular drift and a continued increase in T_c_ and perceived exertion occur throughout 2 h of moderate-intensity work in the heat of a 3:1 work-to-rest ratio. Taken together, worker safety recommendations incorporating work/rest cycles protect some, but not all, workers from experiencing excessive hyperthermia during 4 h of work in hot conditions.

Understanding the impact of work/rest cycles on thermal strain would inform employer decisions regarding scheduled rest breaks during work in the heat. From a public health perspective, the impact of work structure on employee health is critical considering the projected increase in extreme weather in the decades to come, requiring workers to endure more severe and prolonged hot weather [13]. Therefore, the purpose of this study was two-fold: (1) to compare the thermoregulatory, cardiovascular, and perceptual responses between the longer and shorter work/rest cycles, and (2) to determine whether the National Institute for Occupational Safety and Health (NIOSH) recommendations for high intensity work in the heat prevent excessive hyperthermia (T_c_ ≥ 38 °C) during work/rest cycles in a hot environment. We hypothesized that participants following a longer work/rest cycle would experience greater T_c_ compared to a shorter work/rest cycle, and that the majority of participants following both work structures would experience excessive hyperthermia. To our knowledge, this was one of the first studies to directly compare the physiological and perceptual impacts of two work/rest cycles of a single work-to-rest ratio during work in the heat. We tested these hypotheses using two variations of a 2:1 work-to-rest ratio in a 36.3 °C, 30.2% relative humidity environment.

## 2. Materials and Methods

**Figure 1:**
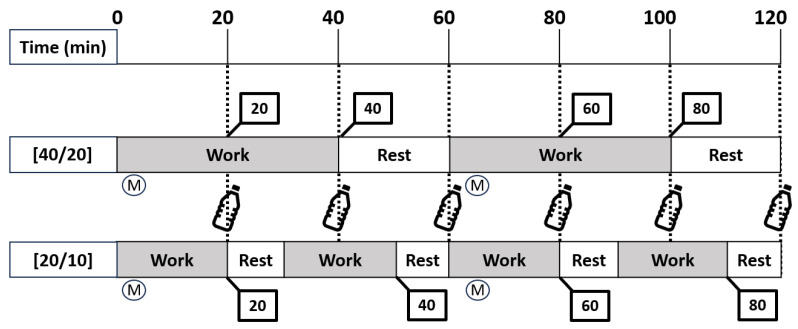
Study design. [40/20] represents the work structure of 40 min of work and 20 min of rest, repeated twice. [20/10] represents the work structure of 20 min of work and 10 min of rest, repeated four times. Numbers in bolded squares indicate comparisons for 20, 40, 60, and 80 min of completed work between trials. Water bottles indicate 237 mL of water consumption. M indicates when metabolic heat production was measured.

This randomized, crossover laboratory study involved three visits: one screening visit and two experimental trials. Trial randomization took place once participants were enrolled in the study, with both the researcher and participant blinded to the trial order during the screening process. To compare responses between two work structures, experimental trials consisted of 80 min of work and 40 min of rest (totaling 120 min) following two different work/rest cycles (Figure 1). Participants completed 2 h of work to a 2:1 work-to-rest ratio structured as either (1) 40 min of work and 20 min of rest each hour [40/20] repeated twice, which follows NIOSH recommendations [14], or (2) 20 min of work and 10 min of rest [20/10], repeated four times. The two experimental trials were conducted with at least three days in between to avoid fatigue and heat adaptations as potential confounders [22]. Females completed the experimental trials during the first ten days of their self-confirmed menstrual cycle to limit the impact on T_c_ [23].

Fourteen (seven females) young, healthy, recreationally active (operationally defined as ≥60 min aerobic activity per week) individuals volunteered to participate in the study (age 24 ± 3 y; height 170 ± 9 cm; weight 72.8 ± 11.0 kg; resting heart rate (HR) 61 ± 8 beats·min^−1^; body fat 20 ± 11%). An a priori power analysis using previous data from our laboratory revealed *n* = 14 was sufficient to see a difference of 0.25 °C between trials. This research was approved by the University at Buffalo institutional review board and all participants were informed of the risks and procedures prior to providing written informed consent and before data collection began. A convenience sample was recruited via email, word of mouth, and flyers placed around campus and in the local community. Participants were excluded from the study if they had cardiovascular, respiratory, renal, neural, or metabolic disorders, musculoskeletal injury affecting exercise, hypertension or tachycardia during screening, a positive pregnancy test at any time throughout the study, current or recent (within the past 6 months) nicotine usage, or reported taking medication known to affect physiologic responses to exercise or thermoregulation. 

During the screening visit, participants completed a medical history and physical activity questionnaire to confirm their eligibility. Resting HR and blood pressure were measured following 5 min of supine rest. Body mass was measured to the nearest 0.01 kg (T51P, Ohaus, Pine Brook, NJ, USA) and body fat percentage was calculated [24] from 3-site (males: chest, abdomen, thigh; females: tricep, suprailiac, thigh) skinfold thicknesses (BetaTechnology Inc., Cambridge, MD, USA) measured in duplicate. Females provided a urine sample to confirm a negative pregnancy status (McKesson Medical-Surgical, Richmond, VA, USA).

Participants not excluded from the study were familiarized with the work protocol to determine the treadmill speed and grade needed to achieve the appropriate intensity. Participants entered the environmental chamber set to the environmental conditions of the trials (36.3 ± 0.6 °C, 30.2 ± 4.0% relative humidity) and were fitted with a two-way breathing mask (Hans Rudolph Inc., Shawnee, KS, USA) connected to a metabolic cart (True One 2400; ParvoMedics, Sandy, UT, USA) calibrated every 2 h to measure oxygen consumption and respiratory exchange ratio to determine metabolic heat production using 1 min averages. Participants began walking at an initial speed of 5.1 kph and a 0% grade for 5 min, after which their internal heat production was calculated [25]. Treadmill speed and/or grade were increased following 3 min stages until metabolic heat production plateaued in the 453–480 W range, which lies at the upper end of “heavy work” as defined in the NIOSH recommendations [14]. Based on the Compendium of Physical Activities [26], this rate of metabolic heat production represents an intensity that is experienced by manual laborers completing high-intensity outdoor work. The final treadmill speed and grade were selected as the starting point for the experimental trials. During the first experimental trial, intensity was confirmed during the first 15 min of work each hour, with adjustments to treadmill speed and/or grade if needed to remain in the intensity range. Any new speeds and grades were applied identically during the second experimental trial.

For experimental trials, participants reported to the laboratory after not exercising or consuming caffeine or alcohol for 12 h prior to the visit. Upon arrival, participants completed a medical history update questionnaire to confirm no changes in medical history that would cause exclusion from the study. Participants provided a urine sample to ensure adequate hydration (USG ≤ 1.025) [27] via urine-specific gravity (USG) (A300CL, Atago, Bellevue, WA, USA), and confirm a negative pregnancy status for females. Participants were then fitted with a chest-strap telemetry HR monitor (Polar Electro, Kempele, Finland) to measure HR every 5 min. Participants self-inserted a rectal thermistor (Medline Industries Inc., Northfield, IL, USA) 12 cm beyond the anal sphincter to measure T_c_ every 5 min and recorded nude body mass in a private room. Four thermochrons (Maxim Integrated, San Jose, CA, USA) were taped to the skin (pectoralis major, deltoid, medial gastrocnemius, quadriceps) to record local skin temperature (T_sk_) as 1 min averages, and calculate mean T_sk_ [28].

Next, participants donned a cotton T-shirt and standard cotton work pants to simulate typical outdoor worker attire, before sitting in the environmental chamber (36.6 °C, 30% RH, no perceptible air movement) for 20 min to equilibrate to the hot conditions. This period represents a worker arriving at a worksite and setting up and planning their work before beginning labor. Following this rest, workers were fitted with breathing masks and baseline values were recorded. Treadmill walking commenced, with the same calibrated treadmill used for all participants throughout both [40/20] and [20/10] trials. Rest breaks consisted of seated rest in the same heated environmental chamber, simulating resting outdoors. Participants reported their perceptions of fatigue on an 11-point scale with 0 = No fatigue at all and 10 = Completely fatigued during work and rest, as used in a previous heat stress study [22]. Rating of perceived exertion (RPE) was measured on an 11-point scale with 0 = Extremely easy and 10 = Extremely hard, and reported during work [29]. Participants consumed 237 mL of refrigerated water (10 °C) every 20 min throughout the protocol, in accordance with NIOSH recommendations [14]. Protocol termination criteria were (1) T_c_ ≥ 40.0 °C, (2) HR ≥ age-predicted maximum HR for five continuous minutes, (3) signs and symptoms of heat illness, and (4) volitional fatigue or request. No participants reached these criteria, with all participants completing the full 2 h of the protocol. After the final rest break, participants towel dried and nude body mass was measured to calculate body mass loss and sweat rate. 

To account for differences in time of work and rest intervals between the trials and to maintain appropriate comparisons between trials, work variables were compared between trials upon completion of 20, 40, 60, and 80 min of work. These time points were selected to align with the end-of-work intervals in the [20/10] trial. Area under the curve for T_c_ was calculated in two ways, T_c_ degree-minutes above resting and T_c_ degree-minutes above 38 °C. Data that violated homogeneity of variance were log transformed prior to analysis. Three-way mixed model analysis of variance (ANOVA) with Greenhouse Geisser corrections and a priori *t*-tests were performed for T_c_, T_sk_, HR, fatigue, and RPE to compare among trials ([40/20], [20/20]), times (20, 40, 60, 80 min of work) and sexes (male, female). Maximum T_c_, T_c_ degree-minutes, percentage change in body mass, sweat rate, and baseline measures were compared between trials using mixed model ANOVA with Greenhouse Geisser corrections and post hoc *t*-tests to compare between trials and sexes. Chi square analysis was performed to compare participants reaching T_c_ ≥ 38 °C in each protocol. Data are reported as mean ± standard deviation. The alpha level was set at 0.05 and all statistics were performed using SPSS version 28 (IBM Corp., Chicago, IL, USA).

## 3. Results

Participants began the trials in a similar physiological state. Baseline USG, T_c_, T_sk_, HR, and nude body mass were not different between trials (all *p* > 0.05) (Table 1). Environmental conditions were also not different between trials (both *p* > 0.05). Participants walked at 3.4 ± 0.1 kph, 4.5 ± 2.0% grade. Internal heat production remained within the desired range of 453–480 W and was not different between trials for either hour 1 ([40/20]: 471 ± 41 W, [20/10]: 455 ± 49 W, *p* = 0.40) or hour 2 ([40/20]: 464 ± 28 W, [20/10]: 453 ± 34 W, *p* = 0.26).

**Figure 2:**
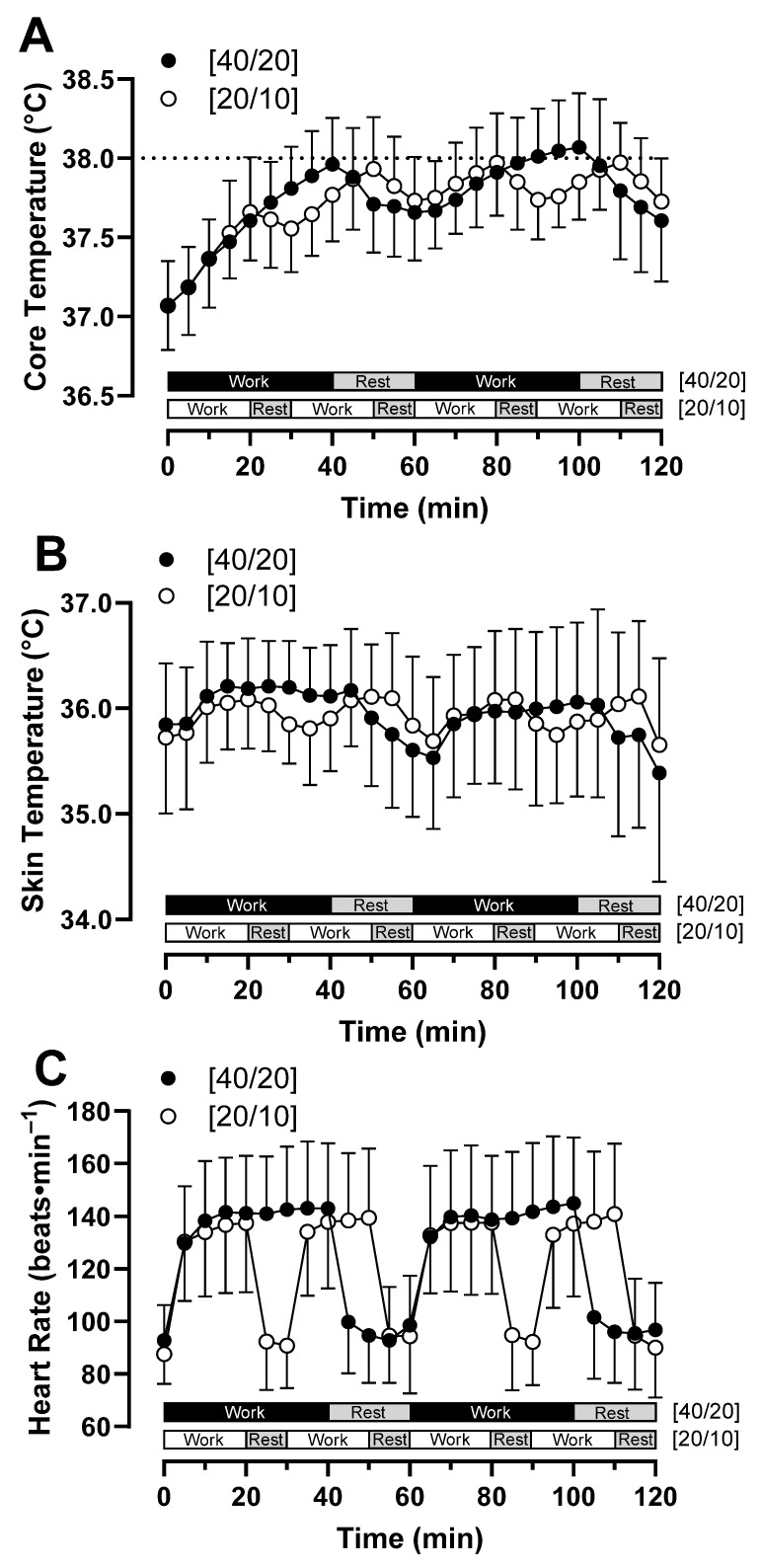
(**A**) Core temperature, (**B**) skin temperature, and (**C**) heart rate responses during [40/20] and [20/10] trials. The horizontal line at a core temperature of 38 °C represents the NIOSH threshold for excessive hyperthermia. *n* = 14.

**Figure 3:**
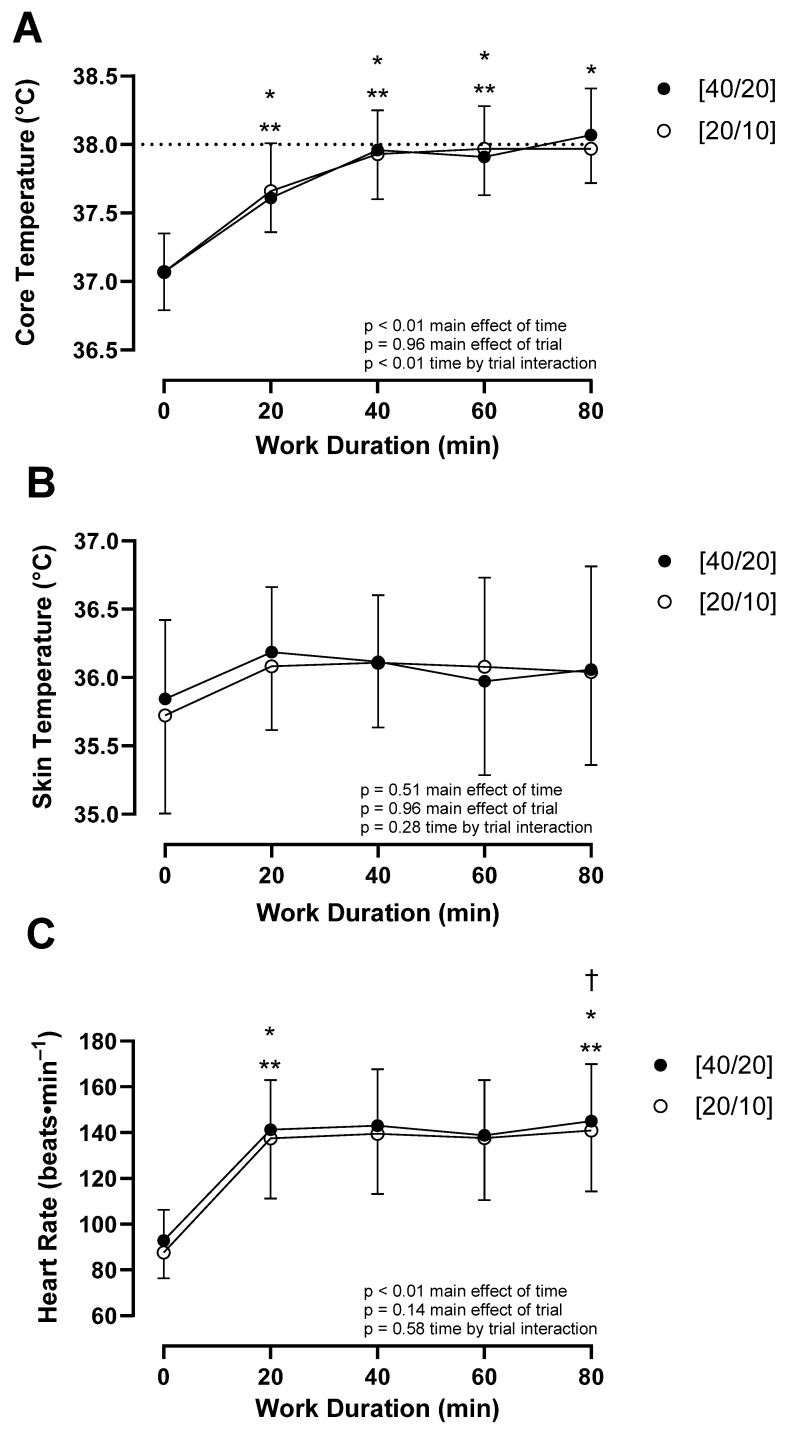
(**A**) Core temperature, (**B**) skin temperature, and (**C**) heart rate responses compared across minutes of work completed during [40/20] and [20/10] trials. The horizontal line at a core temperature of 38 °C represents the NIOSH threshold for excessive hyperthermia. * *p* ≤ 0.05 from previous timepoint in [40/20]. ** *p* ≤ 0.05 from previous timepoint in [20/10]. † *p* ≤ 0.05 between [40/20] and [20/10]. *n* = 14.

**Figure 4:**
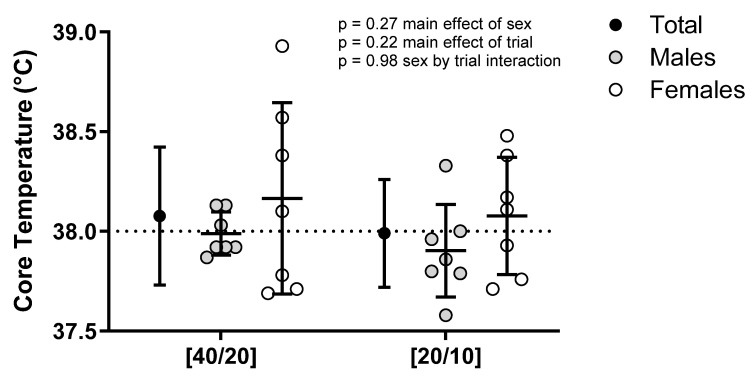
Maximum core temperature during [40/20] and [20/10] trials. The horizontal line at a core temperature of 38 °C represents the NIOSH threshold for excessive hyperthermia. *n* = 14.

T_c_ increased over time (*p* < 0.01) (Figure 2 and Figure 3). A trial by time interaction effect (*p* = 0.01) indicated a difference in T_c_ increase between trials. T_c_ at the end of work during hour 1 was not different between trials (*p* = 0.32), nor was it different between trials following 10 min of rest following this hour of work (*p* = 0.41). T_c_ at end-of-work during hour 2 was not different between trials (*p* = 0.07), nor was it different between trials following 10 min of rest following work this hour (*p* = 0.15). In [40/20], T_c_ was greater at 80 min compared to 60 min of work (*p* < 0.01), while in [20/10], there was no difference at these time points (*p* = 0.93), indicating a plateau in T_c_. Sex did not modulate this relationship (*p* = 0.43). There was no difference in maximum T_c_ between [40/20] (38.08 ± 0.35 °C) and [20/10] (37.99 ± 0.27 °C, *p* = 0.22) (Figure 4), and this relationship was not modulated by sex (*p* = 0.98). 50% (7/14) of participants surpassed the NIOSH excessive hyperthermia threshold during [40/20] compared to 43% (6/14) of participants following [20/10] (*p* = 0.71). T_c_ degree-minutes above resting T_c_ ([40/20]: 81.5 ± 16.3 °C∙min, [20/10]: 79.2 ± 28.1 °C∙min, *p* = 0.66) and T_c_ degree-minutes above 38 °C ([40/20]: 6.8 ± 15.3 °C∙min, [20/10]: 5.0 ± 11.0 °C∙min, *p* = 0.51) were not different between trials, nor were they modulated by sex (*p* = 0.60, *p* = 0.40), respectively.

T_sk_ was not different across time (*p* = 0.51) or between trials (*p* = 0.96), nor was T_sk_ modulated by sex (*p* = 0.39) (Figure 2 and Figure 3). End-of-work T_sk_ ([40/20]: 36.1 ± 0.8 °C, [20/10]: 36.0 ± 0.7 °C, *p* = 0.45) was not different between trials.

HR increased over time (*p* < 0.01) (Figure 2 and Figure 3). HR at the end of the first hour of work was not different between trials (*p* = 0.06), nor was it different following 10 min of rest at the end of this hour (*p* = 0.41). HR at the end of the second hour of work was greater in [40/20] compared to [20/10] (*p* = 0.04), with this relationship maintained following 10 min of rest at the end of this hour (*p* = 0.01). The relationship of HR among trials was not modulated by sex (*p* = 0.23).

**Figure 5:**
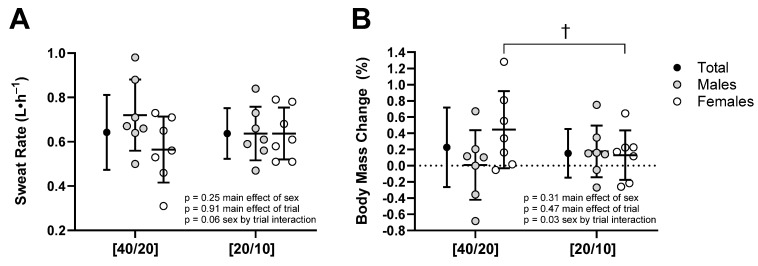
(**A**) Sweat rate and (**B**) percentage of body mass loss during [40/20] and [20/10] trials. † *p* ≤ 0.05 between [40/20] and [20/10]. *n* = 14.

**Figure 6:**
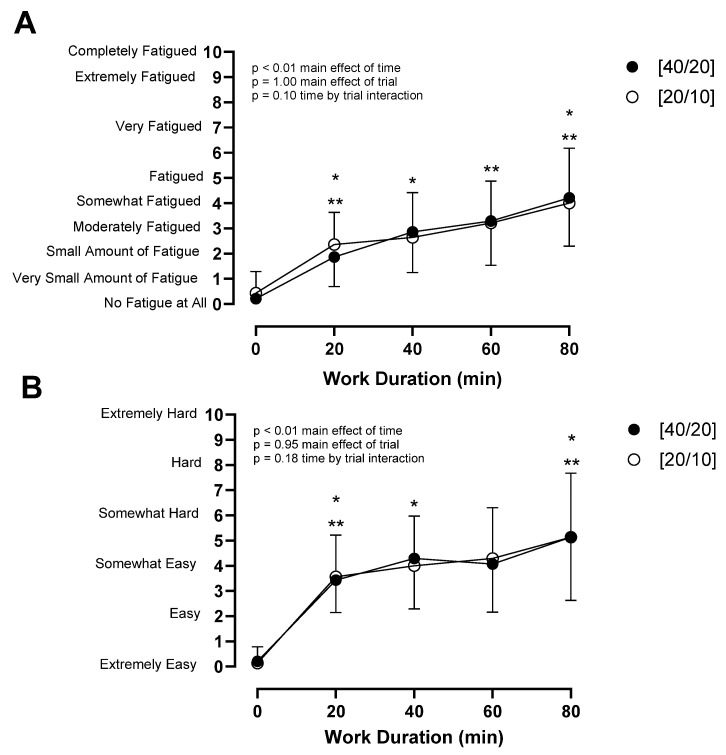
(**A**) Fatigue and (**B**) rating of perceived exertion during [40/20] and [20/10] trials. * *p* ≤ 0.05 from previous timepoint in [40/20]. ** *p* ≤ 0.05 from previous timepoint in [20/10]. *n* = 14.

There was a trial by sex interaction (*p* = 0.03) for percentage change in body mass (Figure 5). Females had a greater percentage of body mass gain in [40/20] (+0.4 ± 0.5%) compared to [20/10] (−0.1 ± 0.3%, *p* = 0.04) while in males, percentage change in body mass was not different between [40/20] (+0.0 ± 0.4%) and [20/10] (+0.2 ± 0.3%, *p* = 0.25). Sweat rate was not different between trials in either females ([40/20]: 0.56 ± 0.15 L·h^−1^, [20/10]: 0.64 ± 0.12 L·h^−1^, *p* = 0.18) or males ([40/20]: 0.72 ± 0.16 L·h^−1^, [20/10]: 0.64 ± 0.12 L·h^−1^, *p* = 0.14). Fatigue and RPE increased over time (*p* < 0.01, *p* < 0.01), but were not different between trials (*p* = 1.00, *p* = 0.95), nor were they modulated by sex (*p* = 0.57, *p* = 0.13), respectively (Figure 6). 

## 4. Discussion

This study examined the thermoregulatory and perceptual responses to two work/rest cycles of a 2:1 work-to-rest ratio in the heat. Our hypothesis was that working in accordance with the shorter work/rest cycle would result in lower T_c_ compared to the longer work/rest cycle, and that both work structures would result in at least half of the participants experiencing excessive hyperthermia. In contrast to our hypothesis, maximum T_c_ was not different between structures. However, the pattern of T_c_ responses differed between trials, with [40/20] resulting in a continued increase in T_c_ during the second hour of work while [20/10] resulted in a plateau in T_c_. Both work structures resulted in at least half of participants exceeding the excessive hyperthermia T_c_ threshold of 38 °C.

Although the majority of our participants reached T_c_ ≥ 38 °C in both protocols, T_c_ plateaued in [20/10] and continued to increase in [40/20] during the second hour of work. This indicates that if work were to continue, it is likely that greater T_c_ would be observed with the longer work/rest cycles compared to shorter work/rest cycles. Mulholland et al. [21] demonstrated similar findings with participants performing treadmill walking and seated rest to a 45/15 min work/rest cycle in a 34 °C, 56% RH environment. T_c_ had a continued increase throughout 2 h of work, despite 15 min rest breaks each hour, indicating that work beyond 2 h would likely result in a further increase in T_c_. However, a limitation of this and previous studies was that an extended protocol was not completed to confirm this hypothesis. If this continued T_c_ increase were to occur, workers would be in a sub-optimal hyperthermic situation. This is particularly concerning for the 43% of males and 57% of females in the present study who displayed a T_c_ greater than 38 °C by the end of work during [40/20], which represents the NIOSH recommendations for work in the heat, echoing previous work [1,18,20,21]. Specifically, females had a large variability in T_c_ responses in [40/20], having a maximum T_c_ range of 37.7 °C to 38.9 °C. There was not as much variability in [20/10], indicating that shorter work/rest cycles could result in more consistent responses. Females in the present study completed trials during the first 10 days of their menstrual cycle. Further work is needed to determine the influence of the menstrual cycle on thermoregulatory responses to work/rest cycles in the heat.

One potential explanation for this finding relates to changes in blood pressure during rest periods. Changes in blood pressure influence body cooling following exercise heat stress [30,31,32]. An upright seated posture during rest, as implemented in the present study, promotes blood pooling in the lower extremity by way of gravity and removes the skeletal muscle-pump to encourage venous return. This therefore reduces cardiac filling, which in turn reduces the amount of blood available to distribute to the skin for heat dissipation. Combined with post-exercise hypotension, this could lead to a slower rate of heat dissipation during rest periods. Given that participants spent more continuous time in this upright seated position during rest breaks in [40/20] compared to [20/10], it is possible that prolonged blood pooling and subsequent decrease in cardiac filling led to the attenuation of heat loss through peripheral vascular beds. While this is plausible, mean arterial pressure during rest breaks was not measured. It stands to reason that the greater end-of-work HR in [40/20] (145 b·min^−1^) compared to [20/10] (141 b·min^−1^) and 6 b·min^−1^ greater HR following 10 min of rest in [40/20] compared to [20/10] could reflect compensatory increases in HR to counteract an extended reduction in mean arterial pressure with longer rest breaks. Future research is warranted to examine changes in mean arterial pressure during rest following work in the heat and the implementation of interventions that promote the maintenance of blood pressure such as supine rest or light active rest such as ankle movement.

The aim of the NIOSH recommendations is to protect workers from excessive heat strain by preventing T_c_ from exceeding 38 °C [14]. Under the conditions of the present study, the safety recommendations failed to protect many workers from excessive heat strain, although varied responses were observed. These results align with others who have shown that heat balance is not achieved when adhering to ACGIH TLV recommendations [19] of 3:1, 1:1, and 1:3 work-to-rest ratios across various environments [18,20,21]. This is not to say that work/rest cycles are not beneficial in mitigating hyperthermia. It is clear that the use of work/rest cycles compared to continuous work extends work duration at a healthy T_c_. Repeated work/rest cycles with greater volumes of rest prevented nearly all young participants from experiencing excessive hyperthermia by the end of 2 h of work while continuous or shorter volumes of rest demonstrated greater proportions of participants with T_c_ ≥ 38 °C [18]. This pattern was echoed in older workers completing a 1:1 compared to 3:1 work-to-rest ratio or continuous work [20]. However, when these data were extrapolated to project T_c_ responses to 4 h of work, the majority of younger and all older participants completing work at 3:1 and 1:1 work-to-rest ratios exceeded a T_c_ of 38 °C, compared to only 25% of younger workers in a 1:3 condition, implying that greater overall rest is beneficial at combating T_c_ increases [18,20]. It should be remembered that the present study investigated one work-to-rest ratio (2:1) in one environmental condition (36 ± 1 °C, 31 ± 4% relative humidity) and compared trials with equal volumes of rest (40 min), so we must be cautious directly comparing these studies. Additionally, although previous studies corroborate our current findings, the work performed was of a lower intensity than what we had participants perform. The aforementioned findings combined with our own challenge the current work/rest cycle recommendations and their ability to prevent excessive heat strain in workers performing moderate to heavy-intensity work. It should be remembered that only select work/rest cycles were performed in one environmental condition, with others remaining unexplored. Additionally, the current protocol consisted of primarily lower body work (i.e., treadmill walking) which may not be representative of responses to whole body or upper body work. Additional study of various work/rest cycles in various environments with a variety of exercise modes warrants investigation. Employers should be aware that the potential for excessive hyperthermia exists, even while following work/rest cycle guidance, and should therefore employ additional heat mitigation strategies such as hydration recommendations, body cooling, and employee education.

In addition to maximum T_c_ reached, time above 38 °C is also an important factor to consider for a more holistic view of a hyperthermic individual. More time spent at a T_c_ ≥ 38 °C represents a greater total thermal load. In the present study, neither the total degree-minutes nor degree-minutes above 38 °C were different between work/rest cycles following 2 h of work. Considering the continued increase in T_c_ in [40/20], future studies should investigate the impact of longer work durations that represent a full workday (e.g., 6–8 h) on total thermal load in the context of degree-minutes above 38 °C.

While the studies mentioned above show that recommended work/rest cycles are generally effective at keeping T_c_ below 38 °C during intermittent work lasting 2 h or less in younger populations, when work duration is extended beyond 2 h, these work structures become less effective. In a more practical sense, many jobs performed in hot conditions are not capped at 2 h of work and therefore may leave workers more vulnerable to negative health and work consequences of excessive hyperthermia, including cardiovascular events [9,10], heat illnesses [5,6], workplace injury [7,8], fatigue [33,34,35], and decreased work productivity [3,4] even when following the recommended work/rest cycles. Therefore, studies investigating intermittent work in hot conditions beyond 2 h, examinations of alternative work/rest cycles, and creating models to accurately predict physiologic responses to heat stress are all necessary to best protect workers and maintain productivity. Another suggestion is to consider that structured work and rest is only one of several potential hyperthermia mitigation strategies that could be used in tandem. Ensuring structed rest breaks along with adequate opportunities for hydration, body cooling, and potentially heat acclimatization are essential for worker health.

Considering that these worker safety recommendations fail to protect many young, active participants without comorbidities from excessive heat strain, it should be expected that other worker populations will also experience excessive hyperthermia, potentially to a greater degree. Lamarche et al. [20] found that nearly all older adults (>50 y) performing moderate intensity work in the heat while adhering to ACGIH TLVs experienced excessive hyperthermia within 2 h of work. When their findings were extrapolated to predict responses to 4 h of work, 100% of participants would have exceeded the 38 °C threshold. In a younger worker population, age-related alterations in thermoregulation have been described to occur as young as 40 years old [36], indicating impaired heat dissipation mechanisms with aging [37,38]. Similarly, clinical populations such as workers with metabolic disorders, hypertension, and obesity are at increased risk of excessive hyperthermia when working in the heat [39,40]. Further research is needed to refine heat strain prevention and intervention strategies for these common worker populations to mitigate negative health consequences and inform NIOSH recommendations.

## 5. Conclusions

We sought to test the hypotheses that participants following a longer work/rest cycle would experience greater T_c_ compared to a shorter work/rest cycle, and that the majority of participants following both work structures would experience excessive hyperthermia. We determined that adhering to a shorter work/rest cycle creates a plateau in T_c_ within 2 h of work in the heat, in contrast to a longer work/rest cycle causing a continued increase in T_c_. Workers completing more than 2 h of work in the heat should consider implementing more frequent rest breaks to mitigate hyperthermia when possible. Current NIOSH recommendations for work in hot environments do not prevent the majority of workers from excessive hyperthermia in the conditions presently studied, indicating a need to improve worker safety recommendations to prevent negative health consequences due to work in the heat.

## Figures and Tables

**Table 1:** Baseline variables and environmental conditions for [40/20] and [20/10] trials. *n* = 14.

Variable	[40/20]	[20/10]	*p*-Value
USG	1.011 ± 0.006	1.009 ± 0.008	0.45
Core Temperature (°C)	37.07 ± 0.28	37.07 ± 0.28	0.98
Skin Temperature (°C)	35.8 ± 0.6	35.7 ± 0.7	0.56
Heart Rate (bpm)	92 ± 13	88 ± 11	0.25
Nude Body Mass (kg)	72.4 ± 11.3	71.2 ± 11.5	0.30
Room Temperature (°C)	36.1 ± 0.7	36.5 ± 0.5	0.07
Relative Humidity (%)	30.3 ± 1.5	28.8 ± 3.9	0.28

*n* = 14. *p*-value represents comparison between [40/20] and [20/10] trials.

## Data Availability

Data can be shared upon reasonable request to the corresponding author.
